# Importin-7 Mediates Nuclear Trafficking of DNA in Mammalian Cells

**DOI:** 10.1111/tra.12021

**Published:** 2012-11-07

**Authors:** Arjun Dhanoya, Tse Wang, Eli Keshavarz-Moore, Ariberto Fassati, Benjamin M Chain

**Affiliations:** 1 The Advanced Centre for Biochemical Engineering, University College LondonTorrington Place, London, WC1E 7JE, UK; 2 Division of Infection and Immunity, MRC Centre for Medical Molecular Virology, University College LondonCruciform Building, Gower Street, London, WC1 6BT, UK; 3 The Wohl Virion Centre, University College LondonCruciform Building, Gower Street, London, WC1 6BT, UK

**Keywords:** DNA, Importin 7, interferon, **MHC** -**I**, mtDNA, nuclear import, polyplexes, transfection

## Abstract

Eukaryotic cells have the ability to uptake and transport endogenous and exogenous DNA in their nuclei, however little is known about the specific pathways involved. Here we show that the nuclear transport receptor importin 7 (imp7) supports nuclear import of supercoiled plasmid DNA and human mitochondrial DNA in a Ran and energy-dependent way. The imp7-dependent pathway was specifically competed by excess DNA but not by excess of maltose-binding protein fused with the classical nuclear localizing signal (NLS) or the M9 peptides. Transport of DNA molecules complexed with poly-l-lysine was impaired in intact cells depleted of imp7, and DNA complexes remained localized in the cytoplasm. Poor DNA nuclear import in cells depleted of imp7 directly correlated with lower gene expression levels in these cells compared to controls. Inefficient nuclear import of transfected DNA induced greater upregulation of the interferon pathway, suggesting that rapid DNA nuclear import may prevent uncontrolled activation of the innate immune response. Our results provide evidence that imp7 is a non-redundant component of an intrinsic pathway in mammalian cells for efficient accumulation of exogenous and endogenous DNA in the nucleus, which may be critical for the exchange of genetic information between mitochondria and nuclear genomes and to control activation of the innate immune response.

Efficient gene expression following either DNA transfection *in vitro* or direct injection *in vivo* in skeletal muscle implies that exogenous DNA molecules can be uptaken into the cell nucleus 1,2. Nuclear uptake of DNA is conserved in eukaryotes from yeast to mammalian cells. For example, several hundred mitochondrial DNA (mtDNA) sequences of different length have been found integrated in nuclear genomes of humans, primates, rat, Drosophila, higher plants and yeast (*Schizosaccharomyces pombe*) 3. Phylogenetic analyses have shown that the process of mtDNA and chloroplast DNA transfer to the nucleus is ongoing; genetic and biochemical experiments have shown that this process is independent of an RNA intermediate and occurs at high frequency 3. Importantly, mtDNA must be able to go across the nuclear envelope because yeast cells have a closed mitosis 4. Similarly, exogenous DNA is uptaken by nuclei of post-mitotic mammalian cells, albeit at lower efficiency compared to dividing cells 1,2,5. There is also evidence that fragments of cellular DNA from apoptotic cells can transfer horizontally between post-mitotic cells be expressed and contribute to cell transformation 6,7.

These lines of evidence support the concept that eukaryotic cells have an intrinsic ability to transport exogenous and endogenous DNA into the nucleus, which may contribute to the exchange of genetic information and their evolution. Despite the fundamental importance of this phenomenon, and our understanding of nuclear import mechanisms 8, little is known on such intrinstic pathways for DNA transport. Crossing nuclear pore complexes (NPCs) rather than transport to the nuclear envelope appears to be the main limiting factor for DNA gene delivery 9. Hence many groups have attempted, with varying degree of success, to improve the efficiency of DNA nuclear uptake by coupling it to nuclear localization signal peptides 10. Nuclear import of DNA was shown to occur through NPCs and to be dependent on energy, hydrolysis of RanGTP and to be distinct from the classical impα/β NLS-import pathway 11,12. Consistent with these observations, the nuclear import receptor transportin (Trn) was shown to promote nuclear accumulation of exogenous DNA in *in vitro* reconstituted nuclei and in digitonin-permeabilized cells 13, although it is not known if Tnr also stimulates DNA nuclear import in intact cells. Therefore, the question whether a specialized cellular pathway for DNA nuclear import exists and what its function might be remains unanswered.

We have recently reported that importin 7 (imp7) is required for efficient DNA transfection into mammalian cells 14 and imp7 and impβ were the only nuclear transport factors found associated to intracytoplasmic plasmid DNA following transfection 15. Imp7 is a nucleocytoplasmic transport protein related to the impβ family of nuclear transport receptors (NTRs) that binds to RanGTP at the N-terminus and to the NPC 16. Members of the importin β superfamily can act as nuclear import or export receptors (or both) depending on whether they bind or release the cargo in the presence of RanGTP. Nuclear *import* receptors bind their cargos in the cytoplasm and release them in the nucleus upon binding to RanGTP, whereas nuclear *export* receptors bind their cargos in the nucleus in complex with RanGTP and dissociate from them in the cytoplasm upon RanGTP hydrolysis 17. Therefore, Ran is a critical regulator of nuclear import.

Imp7 is an import factor for several nucleic acids-binding proteins, including ribosomal proteins 18, the HIV-1 integrase protein 14,19,20, the glucocorticoid receptor 18,21 and as an heterodimer with impβ, histone H1 22. HIV-1 exploits imp7 to maximize nuclear access of its own reverse transcribed, double-stranded DNA genome 14,19. Furthermore, in association with impβ, imp7 mediates nuclear import of adenovirus DNA 23. Taken together these observations suggest that imp7 may be part of an intrinsic pathway for DNA nuclear import, which is exploited by certain viruses to replicate.

To examine further the role of imp7 in DNA nuclear import, we have tested its activity in digitonin-permeabilized cells and in intact cells depleted of imp7 (imp7 KD). We found that imp7 is indeed part of an intrinsic pathway that promotes nuclear import of exogenous and endogenous DNA in mammalian cells. We also found that the lower efficiency of DNA nuclear import detected in imp7 KD cells results in greater activation of the antiviral innate immune response, suggesting that rapid sequestration of DNA into the nucleus may prevent uncontrolled activation of the innate immune system.

## Results

**Figure 1:**
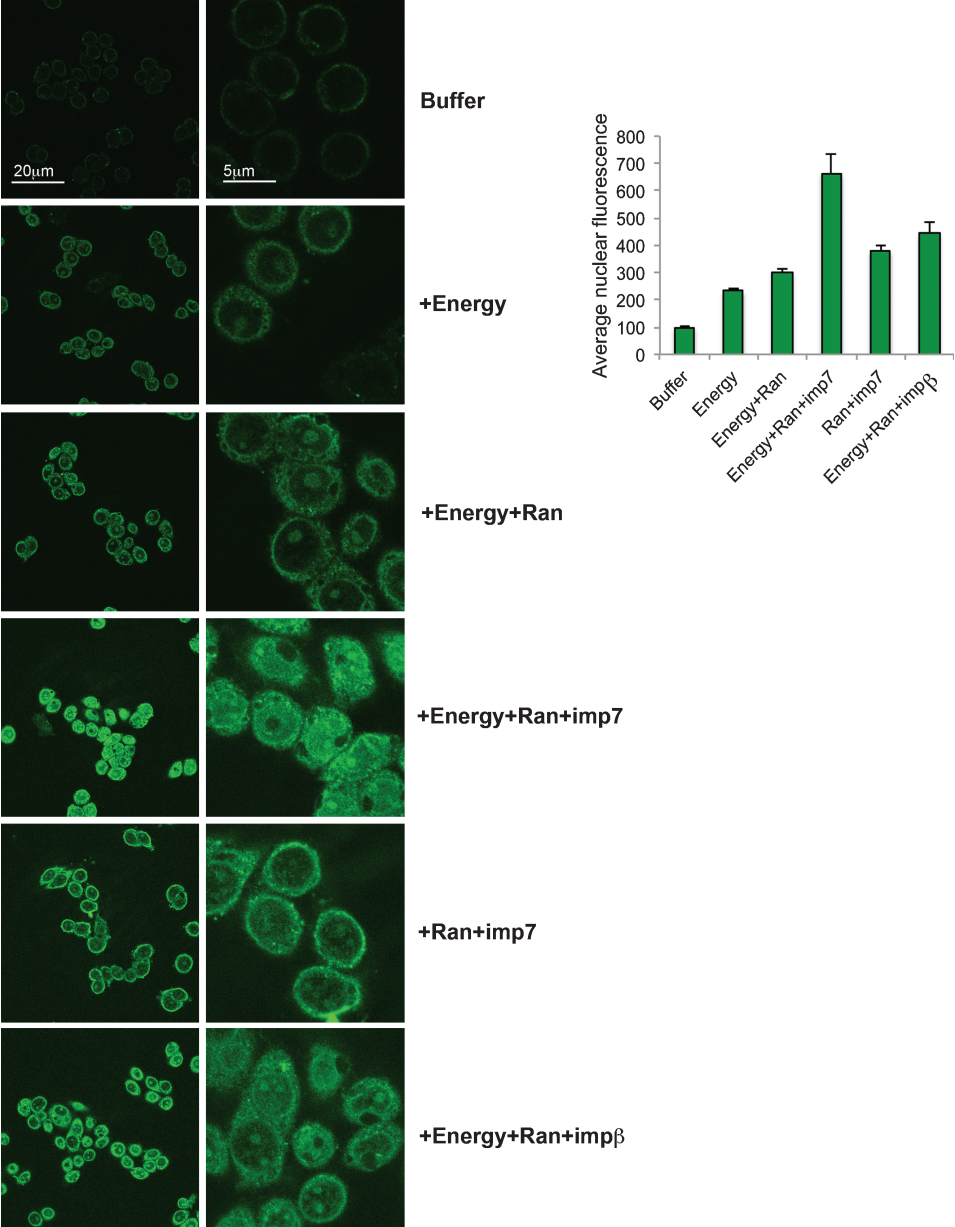
Imp7 stimulates nuclear import of plasmid DNA Nuclear import assay in permeabilized-HeLa cells in the presence of labelled plasmid DNA and: buffer; 1× energy mix (+Energy); 1× energy mix + 1× Ran mix (+Energy + Ran); 1× energy mix + 1× Ran mix + 1 µm imp7 (+Energy + Ran + imp7); 1 µm imp7 + Ran mix (+Ran + imp7); 1× energy mix + 1× Ran mix and 1 µm impβ (+Energy + Ran + impβ). Nuclear import was quantified by ImageJ software and the signal level in the buffer samples is given an arbitrary value of 100. Bars represent mean ± SD from 40–50 cells. Results are representative of four independent experiments.

To directly test if imp7 promotes nuclear import of DNA, we fluorescently labelled a 9.3-kb plasmid DNA 24 and used it in an *in vitro* nuclear import assay. In this assay, cells are permeabilized with digitonin, which selectively solubilizes cholesterol and hence affects the plasma membrane but not the nuclear envelope. Cytosolic contents are removed by washing and nuclear import is reconstituted by the addition of recombinant factors, the Ran system and an energy regenerating system 25. This assay allows to study the contribution of individual components to the import of specific substrates and is widely used in the nuclear import field 12,13,18,19,22. Figure 1 shows that incubation of labelled DNA with an energy regenerating system and with the Ran mix stimulated DNA nuclear accumulation above background, consistent with previous reports 11,12. This background signal is most likely caused by cytosolic factors remaining in the permeabilized cells in sufficient quantity to stimulate low level of nuclear import. However, maximal stimulation of DNA import was observed only when imp7, energy and Ran mix were present together. Energy was required for the process because omitting the energy regenerating system reduced nuclear fluorescence even in the presence of the Ran system and imp7. Impβ on its own did not stimulate DNA nuclear import significantly above that obtained in the presence of energy and Ran (Figure 1). Interestingly, most of the fluorescent signal was detected in nucleoli, however this is not unusual and has been observed before 11,26. Similar results were obtained with different plasmids, thus this effect was not vector specific (not shown). These data are entirely consistent with the lower transfection efficiency seen in imp7 KD cells 14 and indicated that imp7 promoted plasmid-DNA nuclear import in a Ran- and energy-dependent way.

**Figure 2:**
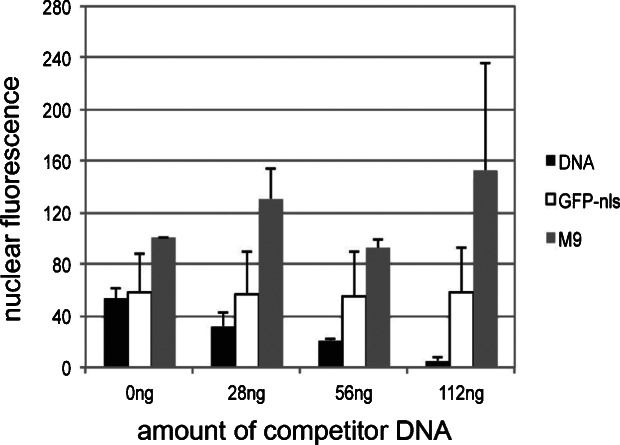
Selective competition of plasmid DNA nuclear import Nuclear import assays in permeabilized-HeLa cells in the presence of fluorescently labelled plasmid DNA (12.5 ng), 1 µm imp7, 1× energy and Ran mix and the indicated amounts of unlabelled plasmid DNA. Experiments were performed in parallel in the presence of 1 µm fluorescent MBP-M9 (M9) or GFP-NLS, 1 µm transportin or impα/β heterodimer, 1× energy and Ran mix and the indicated amounts of unlabelled plasmid DNA. Nuclear import was quantified using the Metamorph software. M9 import in the absence of competitor DNA is given an arbitrary value of 100. Values represent the average fluorescent intensity inside the nucleus relative to control (no DNA) ± SD of three independent experiments.

If imp7 is part of an intrinsic pathway for DNA nuclear import, then this pathway should be inhibited specifically by competitor DNA. To test this prediction, nuclear import assays were performed in the presence of imp7, Ran and energy mix, a fixed amount of labelled plasmid DNA and increasing amounts of unlabelled competitor DNA (Figure 2). To test for the specificity of the assay, parallel experiments were carried out in the presence of Ran and energy mix, transportin and a constant amount of labelled maltose-binding protein (MBP) fused to the M9 peptide of hRNP A1, which binds to and is imported by transportin 27 or a green fluorescent protein (GFP) fused to the classical nuclear localizing signal (NLS) in the presence of the impα/β heterodimer, which binds directly to the NLS 17. Quantification of nuclear fluorescence revealed that competitor DNA inhibited nuclear import of fluorescently labelled DNA in a dose-dependent way but had no effect on the import of MBP-M9 or GFP-NLS (Figure 2).

### Imp7 supports nuclear import of mitochondrial DNA

**Figure 3:**
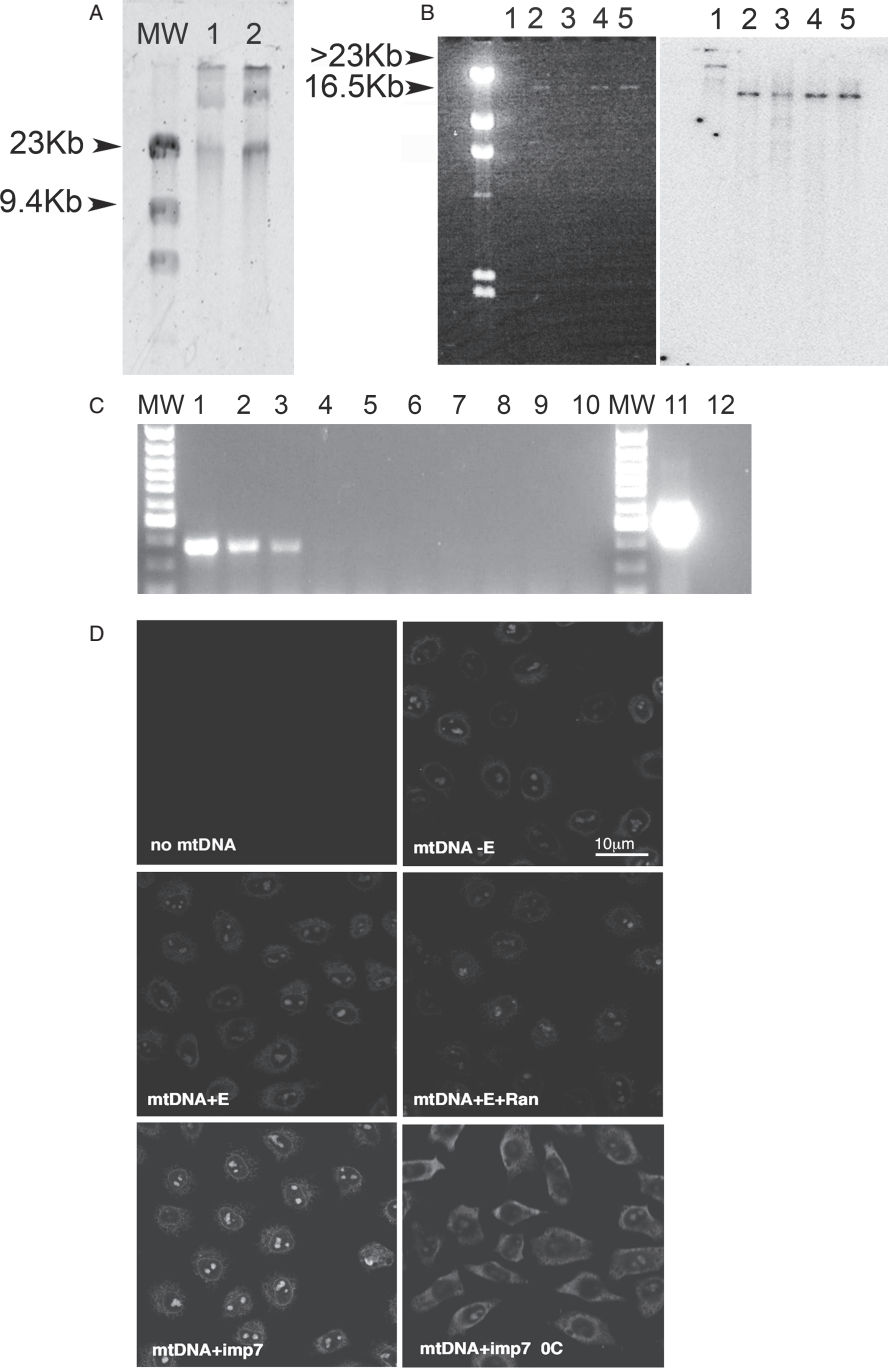
Imp7 induces nuclear import of human mtDNA A) mtDNA was obtained from purified HeLa cells mitochondria after ultracentrifugation through a 4.5 m CsCl gradient and run on a 0.8% agarose gel. The band migrating with an apparent molecular weight of >23 kb was excised and eluted. MW, molecular weight markers; lanes 1 and 2, two different mtDNA preparations. B) Gel-fractionated mtDNA was analyzed by Southern blot with a COX I specific probe. Left panel, agarose gel after ethidium bromide staining; right panel, Southern blot. Lane 1, undigested mtDNA; lane 2, mtDNA cut with BamHI; lane 3, mtDNA cut with ClaI; lane 4, mtDNA cut with PvuII, lane 5, mtDNA cut with XhoI. These enzymes cut once in the mtDNA sequence 33. C) Purified mtDNA analyzed by PCR with primers specific for 28S rDNA or for COX I. MW, molecular weight markers, lanes 1–5, serial dilutions of genomic DNA from 30 to 0.3 ng amplified using 28S rDNA specific primers; lanes 6, no genomic DNA; lanes 7–10, serial dilutions of purified mtDNA from 30 to 1 ng; lane 11, mtDNA (1.25 ng) amplified with COX I specific primers; lane 12, no mtDNA. D) Nuclear import assay into permeabilized HeLa cells in the presence of 20 ng labelled mtDNA and: buffer (−E); 1× energy mix (E); 1× energy mix + 1× Ran mix (E + Ran); 1× energy mix + 1× Ran mix + 1 µm imp7 (imp7). Results are representative of two independent experiments. Scale bar = 10 µm.

Next we sought to test if imp7 promoted nuclear import of mtDNA. Human mtDNA is 16.5 kb in size and hence it was assumed to be too large to be efficiently transported into the nucleus as uncompacted DNA 28,29. However, sequencing of the human genome revealed hundreds of mitochondrial pseudogenes integrated into the nuclear DNA 30. In fact, it is now clear that DNA is transferred from mitochondria to the nucleus at high frequency in eukaryotes 3 and phylogenetic analyses demonstrated that endosymbiotic gene transfer is continuous to this day 31,32. We thus hypothesized that imp7 could mediate nuclear import of mtDNA and hence be part of an endogenous cellular pathway driving DNA transfer from organelles to the nucleus. To directly test this, mitochondria were isolated from HeLa cells and mtDNA purified by CsCl centrifugation (Figure 3A). To control for the quality of the purification procedure, mtDNA was gel purified and digested with four different restriction enzymes known to cut once in the human mtDNA sequence 33 (Figure 3B). The identity and integrity of the purified mtDNA was then confirmed by Southern blotting (Figure 3B). Contamination of mtDNA samples with genomic DNA was examined by PCR with primers specific for the 28S ribosomal DNA sequence and found to be less than 1% (Figure 3C).

The ability of imp7 to mediate mtDNA nuclear import was then tested. mtDNA was fluorescently labelled and used in the nuclear import assay as described. Addition of energy and the Ran mix modestly stimulated nuclear accumulation of fluorescently labelled mtDNA. However, addition of imp7 together with Ran and energy mix stimulated mtDNA nuclear import quite dramatically (Figure 3D). Incubation of the samples on ice blocked mtDNA nuclear import (Figure 3D), which was indicative of an active process. Together, results shown in Figures 2 and 3 support the notion that imp7 is part of an intrinsic cellular pathway stimulating nuclear accumulation of exogenous and endogenous DNA.

### Imp7 is required for nuclear accumulation of plasmid DNA in intact cells

**Figure 4:**
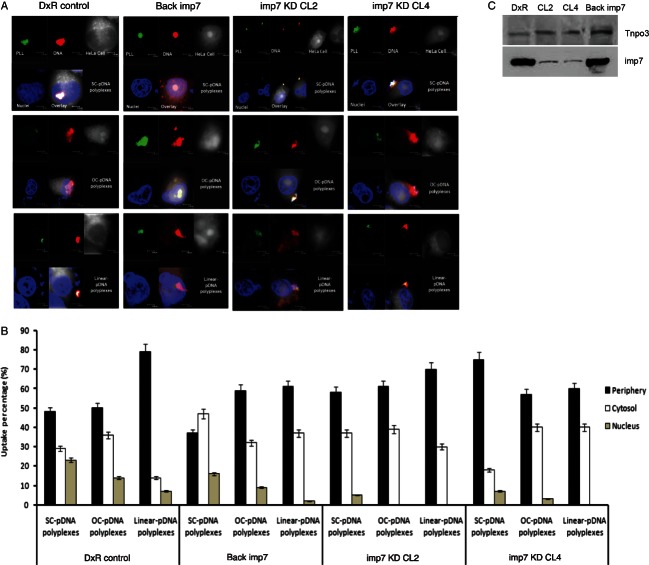
DNA/PLL polyplex nuclear import is reduced in two stable Imp7 KD cell clones (CL2 and CL4) compared to control KD cells (DxR control) and imp7 back complemented cells (Back imp7) Polyplexes (containing 2 µg DNA) were prepared at an equal surface charge at ratios of +1.6 (for SC- and OC-pDNA) and +5 for linear pDNA. A) Confocal microscopy analysis of polyplexes uptake at 1 h post-transfection. PLL is labelled green, DNA is labelled red, the nucleus is labelled blue and the cell cytoplasm is labelled grey. B) Polyplex confocal images were quantified in the periphery, cytosol and nucleus using IImageJ. The figure shows the mean and standard error (SE) of three independent experiments. C) Western blot with an anti-imp7 antibody and an anti-transportin 3 (Tnpo3) antibody (as a specificity control) on total cell extracts obtained from DxR HeLa cells, imp7 KD clone 2 (CL2) and clone 4 (CL4) and Back-imp7 cells used in the experiments.

The nuclear import assay is a powerful technique to investigate individual components of a nuclear import pathway, however it remained critical to confirm the role of imp7 in DNA transport in intact cells. To this end, DNA nuclear uptake was analysed in HeLa cell clones stably depleted of imp7 by expressing an shRNA (hereafter called Imp7 KD clone 2 [CL2] and clone 4 [CL4]), and control cells that either expressed an shRNA targeting the *Discosoma corallimorpharian* DsRed mRNA (DxR) or back complemented cells expressing an imp7 cDNA with silent mutations to make it insensitive to the shRNA targeting imp7 (Back imp7) 14. The level of protein depletion was examined by western blot and found to be ≥90% of control Figure 4). Because naked plasmid DNA does not enter cells efficiently, it was mixed with poly-l-lysine (PLL) to form polyplexes, which could then be transfected. Polyplexes were formulated at differing charge ratios (ratio of PLL to DNA) to optimize the efficiency of DNA uptake (34 and complexes containing plasmid DNA of differing topologies were studied. These included supercoiled (SC), open circular (OC) and linear-DNA complexes 34. DNA was fluorescently labelled with TOTO-3, while PLL was labelled with Oregon Green 488, thus allowing independent monitoring of both polyplex components. HeLa nuclei were visualized by DAPI staining and the cytoplasm was detected by CellMask staining. One hour after transfection, cells were analyzed by confocal microscopy and the level of nuclear and cytoplasmic fluorescence quantified. Polyplexes were classified as being within the cell periphery, cytosol or nucleus as described 33. Polyplexes containing DNA were clearly detected within nuclei and nucleoli of DxR and Back imp7 control cells at 1 h post-transfection (Figure 4). Furthermore, SC and OC DNA forms were transported to the nucleus more efficiently than linear DNA forms, in agreement with a previous study 34. In imp7 KD cells, however, most of the fluorescent signal was extranuclear, and essentially no OC and linear DNA containing complexes were detected in the nuclei of imp7 KD CL2 cells (Figure 4). These data therefore complement the results obtained using the nuclear import assay.

To analyse the kinetics of plasmid-DNA nuclear import, we performed live-cell imaging in transfected cells (Figure S1). Cells were visualized starting immediately after transfection (0 min) and images were acquired every 2 min for 60 min in series of ten stacks. DNA SC polyplexes were detected in the nuclei (and nucleoli) of DxR and Back-imp7 cells as early as 10–14 min post-transfection (Figure S1). DNA accumulation in the nuclei of imp7 KD cells could not be detected 60 min after transfection (the time course of the experiment), and even in the cytoplasm the signal was lower (Figure S1).

**Figure 5:**
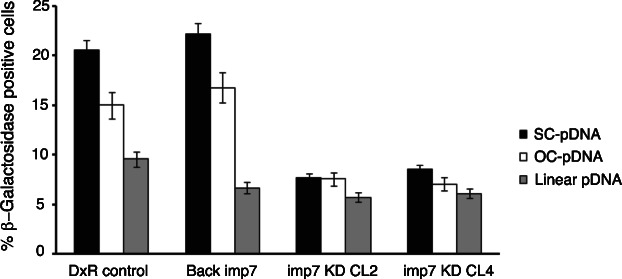
The effect of Imp7 on polyplex gene expression (containing 20 µg DNA) after transfection into control (DxR), imp7 back complemented cells (Back imp7) and two stable Imp7 knockdown clones (imp7 KD CL2 and CL4) β-Galactosidase gene expression was measured 48 h post-transfection. The bar graphs show the mean ± SE of three independent experiments (*p* < 0.05). SC-pDNA, polyplexes of supercoiled plasmid DNA; OC, polyplexes of open circular plasmid DNA; Linear pDNA, polyplexes of linear plasmid DNA.

The plasmid analysed in Figure 4 encodes the gene for β-galactosidase under the control of the cytomegalovirus promoter. We therefore measured transgene expression levels in transfected control and imp7 KD cells and found that imp7 KD had significantly fewer cells expressing the β-galactosidase compared to DxR and Back-imp7 cells (Figure 5). Therefore, there is a close correlation between plasmid-DNA topology and the amount of polyplexes detected in nuclei by confocal microscopy and transgene expression levels.

### Efficient plasmid-DNA nuclear import reduces innate immune responses in transfected cells

Exogenous and endogenous DNA in the cytoplasm can be recognized by several specialized pattern recognition receptors 34–37 and trigger an innate immune response as part of a defence mechanism against invading pathogens. We assessed this response in imp7 KD clones 2 and 4, DxR control and Back-imp7 HeLa cells by measuring the induction of transcription of interferon-induced protein with tetratricopeptide repeats-2 (IFIT2), which acts as a sensitive biomarker for the type I interferon (IFN) response 38.

**Figure 6:**
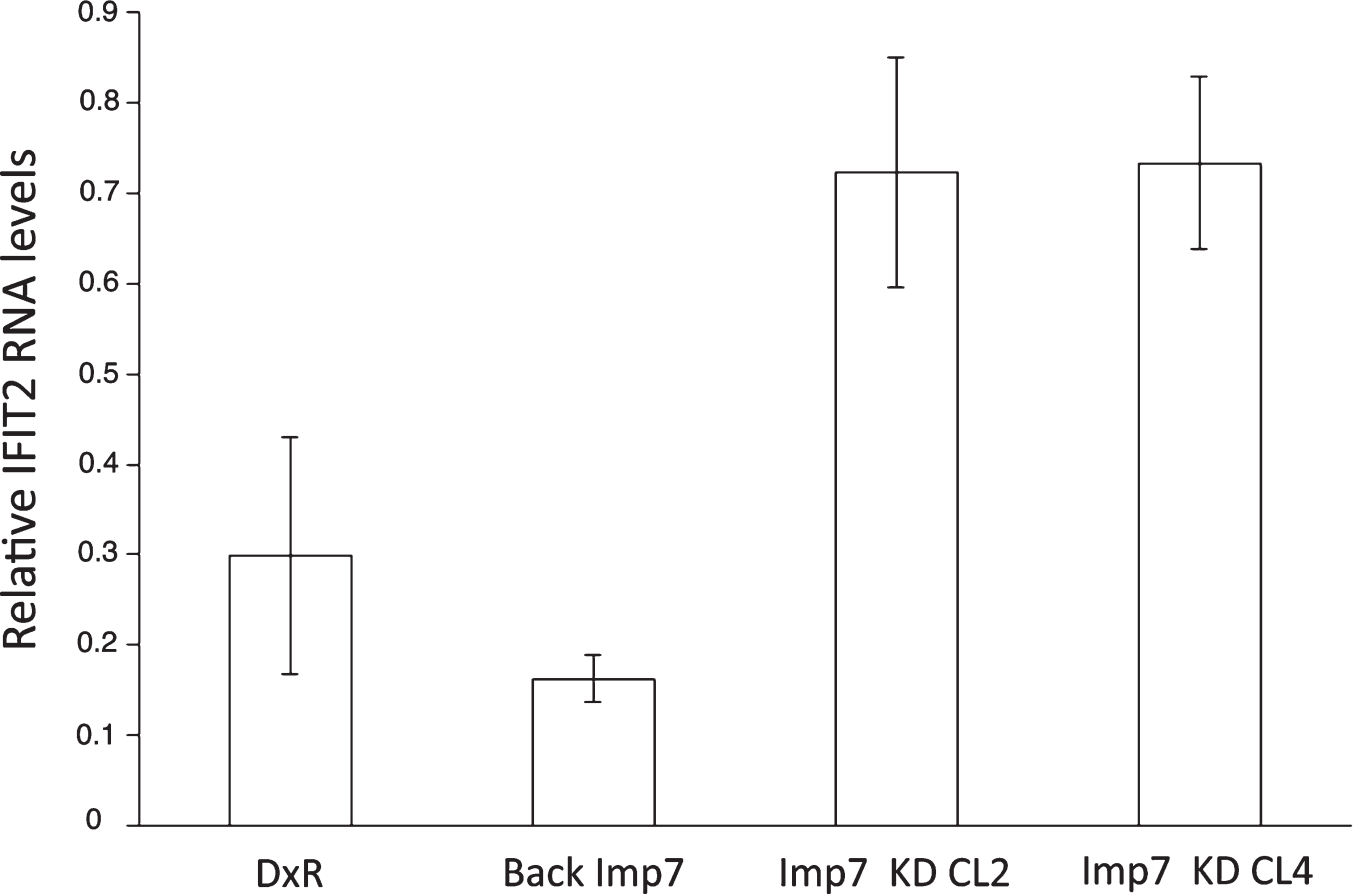
The effect of Imp7 on innate immune responses to transfected DNA The normalised levels of IFIT2 messenger RNA (relative to GAPDH; mean ± SE, *n* = 3) after transfection of DNA polyplexes (containing 2 µg pDNA) were measured in control (DxR), imp7 back complemented cells (Back imp7) and two independent stable Imp7 knockdown clones (imp7 KD CL2 and CL4). The figure shows the results of one representative experiment out of four independent experiments. Gene expression in untransfected cells, or in cells transfected with polylysine alone was undetectable.

Transfection with polyplexes induced IFIT2 gene expression which was quantified by polymerase chain reaction (qPCR). Interestingly, induction of IFIT2 in the Imp7 KD clones was consistently two- to fourfold higher than in the control or Back-imp7 HeLa transfectants (Figure 6). Thus accumulation of cytoplasmic vector DNA in the absence of imp7 activity is associated with an enhanced anti-DNA innate immune response.

**Figure 7:**
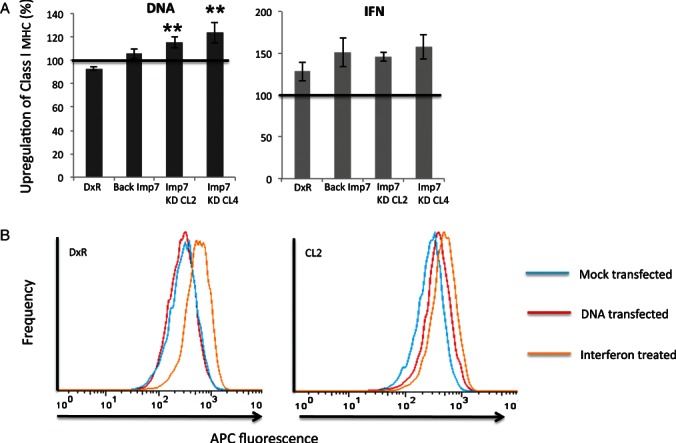
The effect of imp7 on MHC class I expression Control (DxR), back complemented (Back imp7) and two stable imp7 knockdown clones (imp7 KD CL2 and CL4) were transfected with 0.6 µg pHR plasmid or treated with 600 IU/mL IFN-β as shown. MHC class I expression was measured using flow cytometry. A) MHC induction is shown as % compared to mock transfected controls. Horizontal line shows 100% (i.e. no change relative to untransfected). Error bars show standard error of the mean of four independent experiments. Asterisk shows significant upregulation of MHC class I (**p < 0.01, *p < 0.05, Student's *t*-test ). B) A representative flow cytometry histogram plot showing shift in MHC class I expression profile in response to DNA in a imp7 knockdown clone (CL2) but not in a control (DxR) clone.

IFIT2 is an example of a type I IFN induced gene. We also examined the expression of class I MHC on the surface of the HeLa cells, because upregulation of class I MHC is another well-established response to type I IFNs. Control or Imp7 knockdown clones were transfected with pHR plasmid (using Fugene rather than PLL in order to increase transfection efficiency to a level which could be quantified by flow cytometry). Despite a significant background of MHC-I expression in all the cell lines, plasmid transfection reproducibly increased MHC class I expression in the two knockdown clones, but not in control or back complemented HeLa clones (Figure 7). IFN-β induced expression of MHC class I in both control and imp7 knockdown clones equally, indicating that depletion of imp7 did not interfere with the IFN response in these experimental conditions.

## Discussion

Several lines of evidence indicate that eukaryotic cells have evolved specific pathways to transport exogenous and endogenous DNA into the nucleus. Plasmid DNA can access the nucleus of terminally differentiated myotubes 1,2 and it can be observed accumulating into the nucleus of digitonin-permeabilized cells 11–13 or microinjected cells 5,15,26. Furthermore, nuclear transfer of mtDNA and chloroplast DNA occurs frequently in eukaryotic cells, including yeast 3. Because yeast cells replicate through closed mitosis 4, mtDNA must be able to go across the nuclear envelope. Similarly, chromosomal DNA from apoptotic bodies of Epstein–Barr virus transformed B-cells can reach the nucleus of non-dividing cells such as primary human macrophages or bovine aortic endothelial cells, and be expressed 7. However, little is known on the intrinsic pathways for transport of DNA into the nucleus.

Here we showed that imp7 is a critical component of such pathways. Imp7 stimulated DNA accumulation into the nuclei in digitonin-permeabilized cells. The effect was specific because it was observed only when all the components were added, including the Ran system and energy, but not in the presence of imp7 alone. This is consistent with the observations that the Ran system, by inducing disassembly of the import complex on the nuclear side of the NPC, facilitates shuttling of imp7 and allows several cycles of import 17. Importantly, unlabelled DNA could compete nuclear import of labelled DNA in a dose-dependent way but could not compete nuclear import of GFP-NLS or MBP-M9 fusion proteins. Classical NLS depends on the impα/β heterodimer, whereas the M9 peptide depends on Trn for import 17, hence the result that they were not affected by competitor DNA supports the specificity of the DNA-imp7 import pathway. Lack of competition from MBP-M9 was surprising in light of previous results 13, however it is quite possible that DNA import is less efficient than MBP-M9 import and that the affinity of Trn for the M9 peptide is significantly greater than for plasmid DNA. In this case, very large amounts of DNA would be required to outcompete MBP-M9 nuclear import.

Imp7 also stimulated nuclear accumulation of purified human mtDNA in digitonin-permeabilized cells. Considering that human mtDNA has a size of 16.5 kb 33, imp7 is a remarkably efficient DNA import factor. Interestingly, experiments with mtDNA showed a lower background of nuclear import relative to experiments with plasmid DNA (compare Figure 1 with 3), suggesting that residual cytoplasmic contents in digitonin-permeabilized cells were insufficient to support nuclear import of the larger mtDNA molecules. We can exclude that accumulation of fluorescence in the nuclei was caused by diffusion of the fluorescent label from mtDNA into chromosomal DNA, because it was observed only when all the components were added together and not when they were added individually, and it was not observed when samples were incubated on ice.

Results obtained in digitonin-permeabilized cells were complemented by following the fate of transfected DNA polyplexes in intact cells. Lack of imp7 resulted in accumulation of polycomplexes at the periphery and in the cytoplasm of transfected cells and the phenotype was reversed in cells that were back complemented with an imp7 cDNA resistant to the shRNA. In agreement with a previous study 34, supercoiled plasmid DNA transfected into cells was transported into the nucleus most efficiently and very rapidly. Perhaps this should not be very surprising because a single-linear DNA molecule of several kilobase in size can easily span and potentially engage with several NPCs, generating non-productive competition, or may become entangled more easily to other linear DNA molecules, as suggested by earlier studies 35,36. In good agreement with the imaging data showing poor DNA nuclear import in imp7 KD cells, expression levels were also significantly lower in cells lacking imp7 compared to controls, particularly for supercoiled and open circular plasmid DNA. Therefore, inefficient plasmid-DNA nuclear import resulted in inefficient transgene expression, providing a clear functional readout. Because, plasmid transfection mediated by cationic liposomes was also inefficient in imp7 KD cells compared to control cells 14, we conclude that the phenotype is independent of the transfection procedure.

Overall, the imp7 dependency observed in digitonin-permeabilized cells were in excellent agreement with the results of both live imaging and transgene expression. Taken together, these observations show that imp7 is a critical component of an intrinsic pathway for nuclear transport of exogenous and endogenous DNA. This does not exclude that other factors might also be involved 13; in fact this is likely to be the case, given that several cellular nucleic acids-binding proteins may coat plasmid DNA in the cytoplasm. However, imp7 and impβ were the only importins found associated with cytoplasmic plasmid DNA following transfection 15, suggesting that they might have a higher affinity compared to other import factors. The fact that an intermediate level of imp7 depletion did not reduce the efficiency of DNA transfection in a previous study 15 is not surprising: we have also noted that only a substantial depletion (>90%) of imp7 results is a clear-import phenotype 14. Interestingly, the imp7/impβ heterodimer imports histone H1 22, which, as a specialized DNA-binding protein, is a likely candidate to mediate binding of imp7 to DNA. In agreement with this possibility, impβ KD also reduced plasmid-DNA transfection efficiency 15. Because, imp7 is the adaptor protein that recruits impβ to histone H1 22, impβ should not be able to bind to and import DNA on its own. Indeed, this is what we observed in digitonin-permeabilized cells (Figure 1).

Interestingly, HIV-1 also exploits imp7 to promote nuclear import of its pre-integration complex containing the reverse transcribed viral DNA genome 14,19,20 and adenovirus DNA depends on the imp7/impβ heterodimer and histone H1 for nuclear import 23. Some viruses may therefore have evolved to exploit an intrinsic cellular pathway dependent on imp7 in order to traffic viral DNA into the nucleus.

Our work also contributes addressing the fundamental question of what the function of an intrinsic DNA nuclear import pathway in mammalian cells might be. We observed that, in digitonin-permeabilized cells, imp7-induced efficient nuclear import of mtDNA, which is circular and large (16.5 kb) 33. The demonstration that mtDNA can be efficiently transported into the nucleus is conceptually significant, because it supports the hypothesis that mammalian cells have evolved a specialised DNA import pathway. One important function of such a pathway may be to facilitate transfer of genetic material from organelles and also other cells to the nuclear genome, promoting evolution. Exchange between mtDNA, chloroplast DNA and the nuclear genome is known to take place in yeast cells 37, plants 39, primates and humans 31,32, it is still ongoing, occurs in gametes as well as somatic cells 3,31 and is likely to have an important evolutionary significance. Horizontal transfer of chromosomal DNA from apoptotic to bystander cells may also contribute to the evolution of cancer cells 6,7,40.

Our data provide evidence for a possible second function of the imp7-mediated DNA nuclear import pathway: regulation of the innate immune response. DNA from microbial origin and also endogenous DNA are capable of triggering inflammation via recognition of specific receptors, which activate IRF3 and other signalling molecules, and eventually induce type I IFN and pro-inflammatory cytokines 41. Remarkably, most mammalian cell types can recognize intracytoplasmic DNA of a size >25 bp in a sequence and toll-like receptor independent way and activate inflammation 42–44. Lack of specific cytoplasmic nucleases results in accumulation of exogenous and endogenous DNA, triggering uncontrolled inflammation and autoimmunity 43,45. Importantly, endogenous DNA leaked in the cytoplasm induces inflammation in non-immune cells 44 and it has been proposed that sequestration of DNA into the nucleus might be a protective mechanism against uncontrolled innate immune activation 42.

In agreement with previous studies 42,44, we detected a type 1 IFN dependent response upon DNA transfection in HeLa cells, at both RNA and protein level. The induction was consistently greater in cells lacking imp7 and therefore showing a defect of DNA nuclear accumulation. Importantly, the response was not reduced in imp7 KD cells upon addition of recombinant IFN, indicating the IFN pathway was still functional, although we cannot exclude that imp7 may affect the subcellular localization of some specific DNA sensor. Therefore, rapid nuclear import of endogenous and exogenous DNA present in the cytoplasm, such as mtDNA leaked from mitochondria or chromosomal DNA uptaken from apoptotic cells, may be important to prevent uncontrolled activation of the inflammatory response. In support of our model, it has been recently reported that mtDNA escape from autophagy and its prolonged presence in the cytoplasm causes inflammation and heart failure in a mouse model 46.

## Materials and Methods

### Plasmid-DNA preparation, purification and labelling

Plasmids pHR 24 and pSVβ – 6.9 kb (Promega) were propagated within *E. coli* DH5α cells. Plasmids were purified and quantified according to a previous protocol 34. Generation of linear, OC and SC DNA molecules was carried out as previously described 34. Naked pSVβ pDNA was labelled using the nucleic acid fluorescent stains TOTO-3 (Invitrogen) at a final concentration of 4 µm as described 34.

### Production and labelling of PLL/DNA polyplexes

PLL was fluorescently labelled with Oregon Green 488, succinimidyl ester (Invitrogen) as previously described 26. Unbound dye was removed by spin column purification in accordance to the manufacturer's protocol (Invitrogen). Plasmids were complexed with PLL hydrobromide (Sigma) of molecular weight 9600 according to Dhanoya et al. 2011. A total volume of 100 μL was used for polyplexes prior to the addition of cells for transfection, which was performed as previously described 34.

### Cells and transfection

DxR, stable imp7 KD clone 2 and clone 4 were previously described 14. To generate Back-imp7 cells, two silent mutations were introduced into the human importin 7 cDNA (Origene) using the QuickChange II XL site directed mutagenesis kit (Stratagene) with primer CCTCGAAAAAAAGATGG**T**GCCCTGCA**C**ATGATTGGC (mutations in bold). The plasmid was cotransfected with a Hygro^r^ pUC plasmid into HeLa imp7 KD clone 4 and cells selected in media containing 500 µg/mL hygromycin B and 1 µg/mL puromycin. Cells were cultured in Dulbecco's modified Eagle's medium (DMEM) (GIBCO®), with the addition of 10% foetal calf serum (FCS), 2 mm glutamine and 1 µg/mL puromycin at 37°C in 5% CO_2_. Transfection was performed using PLL as described 34 or Fugene 14. Briefly, cells were seeded in 6-well plates (2 × 10^5^/well for CL2 and CL4 or 10^5^/well for DxR and BC cells). The next day, cells were transfected using a mixture of 3 μL Fugene 6 and 600 ng DNA diluted in 37 μL serum-free DMEM, according to the manufacturer's instructions. Cells were analysed 24 h after transfection by flow cytometry (pHR) or β-Gal staining (pSV-β-galactosidase).

### Confocal microscopy – fixed cell analysis

A Leica SP2 confocal microscope was used to collect images of cells mounted on the appropriate slides. Fluorescence images were collected using a scan speed of 400 Hz and eight frame averaging. Cells fixed to coverslips were stained following transfection with HCS CellMask™ Stains (Invitrogen) for a period of 30 min according to the manufacture's protocol. The stain displays excitation and emission spectra of 556 and 572 nm, respectively. Nuclei were detected using 4,6-diamidino-2-phenylindole (DAPI) (Vectashield) (excitation: 405 nm, emission: 400–450 nm). DNA was detected via TOTO-3 (dimeric cyanine nucleic acid stains – Invitrogen) (excitation: 642 nm, emission: 660 nm). PLL was detected via Oregon Green 488 (Invitrogen) (excitation: 488 nm, emission 524 nm). To quantify the fluorescent signal ∼30 cells from each slide were randomly chosen under the DAPI filter and the number of cell-associated polyplexes were counted and classified on the basis of their intracellular location (cell periphery, cytosol or nuclei of the respective cell) using ImageJ software. The number of polyplexes within each cellular compartment was expressed as a percentage of the total number of polyplexes counted within the group of 30 cells. The number of cells 29 was selected as this was found to be statistically sufficient for quantification as shown by previous studies 15. Each experiment was repeated three times. Slides were blinded with regard to experimental conditions before counting to reduce possible bias.

### Live-cell imaging

HeLa cells were seeded (∼5 × 10^5^ cells per well) overnight in an 8-well chambered glass coverslip (Lab-Tek™) at 37°C. The following day the media was removed and replaced with RPMI 1640 (1×) media without phenol red (Invitrogen). Cells were incubated with 5 µg/mL Hoechst 34580 nuclear stain (Invitrogen) for 2 h at 37°C. The coverslips were then mounted on a Leica SPE2 inverted microscope with sequential channel capture. The microscope chamber temperature was set at 37°C and fluorescently labelled polyplexes (containing 2 µg DNA) were added to the cells and imaging was carried out immediately to monitor transfection in real time. Images were recorded every 2 min up to 1 h. Slice-by-slice sectioned images of single cells were captured at a thickness of 0.2 µm. A total of ten image slices were taken at each time point. Each frame of the time lapse series was processed on ImageJ software.

### Isolation of human mtDNA

To isolate mitochondria from HeLa cells, a modified version of Bogenhagen and Clayton's protocol was used 1974. Approximately 10^8^ cells were washed once in PBS and resuspended in five pellet volumes of hypotonic buffer (10 mm HEPES [pH 7.9], 1.5 mm MgCl_2_, 10 mm KCl, 5 mm dithiothreitol [DTT], 20 µg aprotinin/mL, 20 µg leupeptin/mL) and centrifuged for 5 min at 600 × g in an Eppendorf microcentrifuge. Supernatant was kept in a separate tube and the pellet was resupended in five volumes of hypotonic buffer and incubated for 10 min on ice. Cells were homogenized with 10–15 strokes in a Dounce homogenizer, quickly resuspended in 1/6 volume of ST buffer (50 mm Tris–HCl pH 7.5, 2 m sucrose, 35 mm EDTA) and centrifuged twice at 600× ***g*** for 10 min each at 4°C to pellet nuclei. The supernatant was layered on the top of a 1.5 m sucrose buffer (5 mm Tris–HCl pH 7.5, 5 mm EDTA) and centrifuged at 80 000× ***g*** maximum for 1 h at 4°C. Mitochondria were collected at the interface using a glass Pasteur pipette and washed twice in 2.5 mL of 10 mm Tris–HCl pH 7.5, 1 mm EDTA and 0.25 m sucrose. The pellet was resuspended in 5 mL lysis buffer (50 mm Tris–HCl pH 8, 5 mm EDTA, 0.4% SDS), digested by protease K treatment (100 µg/mL) at 55°C for 12–16 h and nucleic acids extracted by phenol chloroform and ethanol precipitation. DNA was resuspended in 50 μL TE buffer, layered on the top of a 4.5 m CsCl solution and centrifuged at 353 000× ***g*** for 24 h at 20°C. The faster migrating band was collected, washed several times in water-saturated butanol and dialysed against large volumes of TE buffer at 4°C with two buffer changes. DNA was loaded onto a 0.8% low-melting agarose (USB Corporation) and separated by electrophoresis for 16 h at 30 V in TAE buffer followed by SYBR Gold staining. The band migrating with an apparent molecular weight >23 kb was eluted from the gel by digestion with Beta-Agarase (New England Biolabs) and ethanol-precipitated. The identity of the mtDNA was confirmed by digestion with appropriate restriction enzymes and Southern blot using a COX I specific probe. Briefly, mtDNA was separated onto a 0.8% agarose gel and transferred onto a Hybond N+ membrane (Amersham) by capillary transfer in 0.4 m NaOH. A COX I single-strand specific probe was generated by PCR using the mtDNA reverse complementary primer (see above) and purified mtDNA as template in the presence of ^32^P-dCTP (150 μCi). The membrane was hybridized for 16–18 h, washed twice in 2xSSC at 55°C 30 min each, once in 2xSSC + 0.1% SDS at 55°C for 30 min followed by one final wash in 2xSSC and exposed to a PhosphorImager screen. The signal was visualized by a Storm 860 PhosphorImager (Molecular Dynamics).

### Nuclear import assay

pHR plasmid DNA and mtDNA (30 ng/μL) were labelled with YOYO-1 (Molecular Probes) diluted 1:5000 in import buffer (20 mm HEPES-KOH (pH 7.3), 110 mm potassium acetate, 5 mm magnesium acetate, 0.5 mm EGTA) for 1 h at room temperature in the dark and dialyzed against large volumes of import buffer for >4 h at 4°C in the dark. The nuclear import assay was carried out as previously described 19 in the presence labelled DNA (amount indicated in figure legends), 1 µm imp7 or impβ, 1× Ran mix (2 µm Ran (GDP form), 0.2 µm NTF2, 0.2 µm RanBP1, 0.2 µm RanGAP1) and 1× energy mix adjusted to pH 7.3 (1 mm ATP, 1 mm GTP, 20 mm creatine phosphate, 40 U/mL creatine phosphokinase). Samples were washed three times in import buffer, fixed on ice for 5 min with 2% paraformaldehyde in import buffer and analysed by confocal microscopy. Images acquired by confocal microscopy were analysed by MetaMorph software version 4.5r4 (Universal Imaging Corp.) for quantification. Thresholding was performed to define areas to be analyzed. The threshold value was set so that no objects were visible in negative control samples. Integrated morphometric analysis (total grey value) was performed on the visible objects in the test samples and the total grey value divided by the number of nuclei per field 48.

### RNA extraction

HeLa cells (DxR control, Back Imp7, Cl2 and Cl4 Imp7 KD) were seeded in 6-well plates (∼1 x 10^6^ cells/well) for 48 h. Cells were transfected with polyplexes containing 20 or 0.2 µg pDNA for 4 h (time required to stimulate anti-viral response). Subsequently cells were centrifuged (1400× ***g*** for 5 min), washed and RNA was extracted via the RNeasy Mini Kit (Qiagen) according the manufacturer's instructions. All subsequent reactions were carried out in RNase free conditions. RNA samples were treated with the DNA-*free*™ Kit (Applied Biosystems) according to the manufacturer's instructions. Purified RNA was stored at −80°C.

### RT-qPCR

cDNA template was synthesized using Omniscript® Reverse Transcription Kit (Qiagen) according to the manufacturer's protocol. All subsequent reactions were carried out in DNase free conditions. qPCR was performed in a final reaction volume of 25 μL containing 1× PCR buffer including Taq man polymerase enzyme (Platinum® Quantitative PCR Super Mix-UDG w/ROX, Invitrogen) along with 7.5 µm of each primer (forward, reverse and probe) according to the manufacturer's protocol. Primer sequences were as follows; GAPDH (glyceraldehyde 3-phosphate dehydrogenase) forward: 5′-GGCTGAGAACGGGAAGCTT-3′, GAPDH reverse: 5′-AGGGATCTCGCTCGCTCGCTCGCTCCTGAA-3′, GAPDH probe: 5′FAM-TCATCAATGGAAATCCCATCCCATCACCA-3′ which was fluorescently labelled with 6-carboxyfluorescein (FAM, at a filter setting of 520 nm). To quantify IFIT2, the 1× PCR buffer was supplemented with IFIT2 mix (Applied Biosystems) which contained the IFIT2 forward and reverse primers and probe in a final volume of 25 μL. The qPCR reaction was supplemented with 5 μL cDNA. qPCR was performed on an Eppendorf MasterPlex. Cycle conditions were; 55°C for 2 min, 95°C for 10 min; 95°C for 15 s and 60°C for 1 min for 40 cycles.

### Flow cytometry

At 24 h post-transfection, HeLa cells were detached by trypsin treatment, centrifuged and resuspended in 1% paraformaldehyde in PBS for 15 min. Non-specific binding sites were blocked by incubation in 10% FCS for 30 min. The cells were then incubated with a monomorphic anti-MHC class I antibody-APC conjugate (BD Pharmingen™, Cat. No. 555555) for 1 h, washed and analyzed using a two-laser FACSCalibur (Becton Dickinson).

### Statistical analysis

One-way anova or *t*-test was employed to deduce levels of statistical significance. Level of significance selected was *p* = 0.05.

## Supporting Information

**Figure S1: Kinetics of plasmid-DNA polyplexes nuclear import analyzed by live-cell microscopy.** Uptake of SC-polyplexes in DxR control HeLa cells (A) and Imp7 KD CL2 cells (B). Microscope images were collected as a stack of ten image slices. The figure shows the middle slice (slice number five out of ten) spanning a time course of 60 min. Polyplexes contained 2 µg DNA. Arrows show accumulation of intranuclear DNA.
