# Supplementary material for: Importin-7 Mediates Nuclear Trafficking of DNA in Mammalian Cells
Source: Traffic. 2012 Nov 7;14(2):165–75. doi: 10.1111/tra.12021 (PMC3672689; doi:10.1111/tra.12021)
Supplement: Supplementary file 1 [file tra0014-0165-SD1.doc]

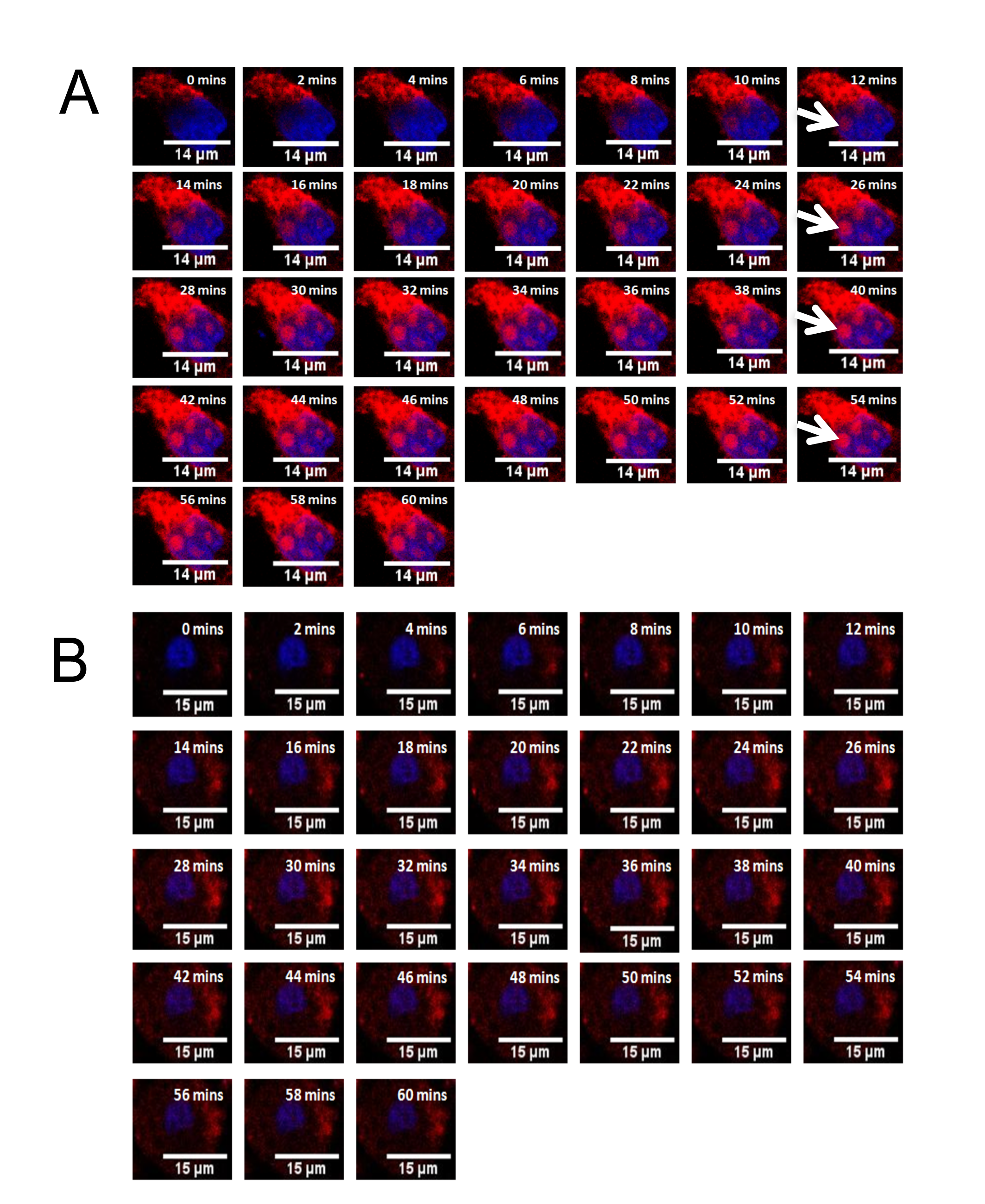


**Supplementary Figure 1**

Kinetics of plasmid DNA polyplexes nuclear import analyzed by live cell microscopy. Uptake of SC- polyplexes in DxR control HeLa cells (A) and Imp7 KD CL2 cells (B). Microscope images were collected as a stack of ten image slices. The figure shows the middle slice (slice number 5 out of 10) spanning a time course of 60 minutes. Polyplexes contained 2µg DNA. Arrows show accumulation of intranuclear DNA.
